# Comparing performance of McGrath MAC videolaryngoscope in morbidly obese and nonobese patients

**DOI:** 10.3906/sag-2001-246

**Published:** 2020-12-17

**Authors:** Ying GAO, Liu-Jia-Zi SHAO, Fu-Shan XUE

**Affiliations:** 1 Department of Anesthesiology, Beijing Friendship Hospital, Capital Medical University, Beijing P. R. China

***Re: Arslan Zİ, Yörükoğlu HU.*** Tracheal intubation with the McGrath MAC X-blade videolaryngoscope in morbidly obese and nonobese patients. Turk J Med Sci 2019; 49(5):1540-1546.

**To the Editor,**

In the recent article by Arslan and Yörükoğlu [1] comparing the performance of the McGrath MAC X-blade videolaryngoscope for tracheal intubation in morbidly obese and nonobese patients, the authors showed that this videolaryngoscope could safely be used both in nonobese and morbidly obese patients, with the aid of some key maneuvers and a statistically significant, but clinically negligible, prolongation of intubation time. Given that difficult airway remains one of the main reasons for adverse outcomes in surgical patients receiving general anesthesia and the videolaryngoscope is one of the most promising first-line tools for difficult airway management [2], their findings have potential clinical implications. However, in order to differentiate the effects of one factor on the primary study endpoint in a randomized controlled trial, all of the other factors must be standardized in order to avoid potential biases. We note several issues in this study that would have made the generalization and interpretation of their findings difficult.

First, a main purpose of this study was to compare the performance of the McGrath MAC videolaryngoscope in morbidly obese and nonobese patients. On the basis of body mass index (BMI), participants were arbitrarily divided into nonobese (BMI <30) and morbidly obese (BMI >35) groups. Indeed, BMI is the internationally accepted standard method for classification of obesity. According to the international criteria of BMI, however, patients are allocated to five different categories: normal, 18.5–24.9 kg/m2; overweight, 25.0–29.9 kg/m2; class-1 obesity, 30.0–34.9 kg/m2; class-2 obesity, 35.0–39.9 kg/ m2; and class-3 obesity, ≥40 kg/m2. Morbid obesity is considered class-2 or -3 obesity plus significant obesityrelated comorbidities [3]. In their study, the mean BMI of nonobese patients was 26.7 kg/m2. That is, the nonobese group included normal patients and those with overweight and class-1 obesity. Thus, we argue, the authors used an inappropriate definition of nonobese patients. In method, moreover, they described a sealed envelope technique that was used for classification of participants. Evidently this is not true, as their study is not a randomized clinical trial. In fact, patients were classed according to BMI, and the sealed envelope technique, which is often used for classification of participants in a randomized clinical trial, was not required.

Second, in this study all patients were placed in the supine position for preoxygenation and intubation. This is not routine clinical practice for morbidly obese patients. It is generally recommended that morbidly obese patients be placed in a 30° head-up position, as this can improve preoxygenation and laryngoscopic view [4]. The improper use of the supine position for morbidly obese patients in this study may be one of main reasons for more requirements of reinsertion and cricoid pressure maneuvers during intubation, a longer intubation time and a higher rate of desaturation in this group. This design limitation would have made generalization of the author’s findings difficult.

Third, we noted that all nonobese and morbidly obese patients were intubated successfully, but most of intubations were completed on the second attempt and required some aiding maneuvers such as slight removal of the device, handling force, use of stylet, 90° anticlockwise rotation, and head flexion. The McGrath MAC videolaryngoscope used in this study has a Macintosh blade, and according to the manufacturer, there is no need of a stylet for intubation. When performing intubation with videolaryngoscopy, however, it can be very helpful for bringing the tube tip up to the glottic opening. The available evidence states that the routine use of a stylet can facilitate intubation with a Macintosh videolaryngoscope, especially for management of difficult airways [5,6]. Thus, we believe that different results would have been obtained if the study design included the routine use of a styletted endotracheal tube.

Finally, in the postoperative care unit, postoperative sore throat was evaluated as a minor complication of intubation. However, the authors did not clearly describe whether duration of anesthesia and intraoperative dosages of opioid drugs were comparable between groups. It is believed that prolonged duration of anesthesia is associated with an increased risk of postoperative sore throat. Furthermore, intraoperative use of opioid drugs can significantly affect the occurrence of early postoperative sore throat [7]. In the absence of a comparison of important risk factors affecting the occurrence of early postoperative sore throat, we argue that the secondary outcome findings and subsequent conclusions should be interpreted with caution, as they may have been determined using an incomplete methodology.

We believe that addressing the above issues will be helpful in preventing any optimistic interpretation or misinterpretation of study results.
